# Nasal nitric oxide in healthy Chinese children aged 6–18 years

**DOI:** 10.3389/fped.2023.990510

**Published:** 2023-05-09

**Authors:** Yufen Wu, Hao Zhang, Jinrong Wang, Yuling Han, Yongsheng Shi, Qiaoling Zhang, Li Shen, Haohua Jiang, Chunmei Jia, Yanyan Yu, Zhen Long, Minghong Ji, Aihong Liu, Chunhong Pan, Dongjun Ma, Jinhong Wu, Fuli Dai

**Affiliations:** ^1^ Department of internal medicine, Shanghai Children's Medical Center Affiliated to Shanghai Jiaotong University School of Medicine, Shanghai, China; ^2^ Pediatric Respiratory Department, The First Affiliate Hospital of Shandong Provincial Medical University, Shandong, China; ^3^ Department of Respiratory, Children's Hospital Affiliated to Shandong University, Shandong, China; ^4^ Department of Pediatric Respiratory, Maternity and Child-Care Hospital of Gansu Province, Lanzhou, China; ^5^ Department of Pediatric Respiratory, Maternal and Child Health Hospital in Inner Mongolia Autonomous Region, Hohhot, China; ^6^ Shanghai Jiao Tong University School of Medicine, Shanghai, China; ^7^ Department of Respiratory Medicine, Shanghai Chest Hospital Affiliated to Shanghai Jiao Tong University School of Medicine, Shanghai, China; ^8^ Department of Pediatric Respiratory, The Fourth Hospital of Baotou, Baotou, China; ^9^ Suzhou Municipal Hospital(Suzhou Hospital Affiliated to Nanjing Medical University), Suzhou, China; ^10^ Department of Pediatric Respiratory Medicine, Maternal and Child Health Hospital of Hubei Province Affiliated to Huazhong University of Science and Technology Tongji Medical College, Wuhan, China; ^11^ Department of Pediatric, The First Affiliated Hospital of USTC (Anhui Provincial Hospital), Anhui, China; ^12^ Department of Respiratory Medicine, Children's Hospital of Shanxi, Taiyuan, China; ^13^ Department of Allergy, Children's Hospital of Urumqi, Urumqi, China; ^14^ Department of Pediatric Respiratory Medicine, Luoyang Maternal and Child Health Hospital, Luoyang, China

**Keywords:** reference values, Chinese children, fractional exhaled nitric oxide testing, fnNO, factor analysis

## Abstract

**Objectives:**

To obtain the normal values of fractional concentration of nasal nitric oxide in Chinese children aged 6–18 years, so as to provide reference for clinical diagnosis.

**Methods:**

2,580 out of 3,200 children (1,359 males and 1,221 females), whom were included from 12 centers around China were taken tests, their height and weight were also recorded. Data were used to analyze the normal range and influencing factors of fractional concentration of nasal nitric oxide values.

**Measurements:**

Data was measured using the Nano Coulomb Breath Analyzer (Sunvou-CA2122, Wuxi, China), according to the American Thoracic Society/European Respiratory Society (ATS/ERS) recommendations.

**Main Results:**

We calculated the normal range and prediction equation of fractional concentration of nasal nitric oxide values in Chinese children aged 6–18 years. The mean FnNO values of Chinese aged 6–18 yrs was 454.5 ± 176.2 ppb, and 95% of them were in the range of 134.5–844.0 ppb. The prediction rule of FnNO values for Chinese children aged 6–11 yrs was: FnNO = 298.881 + 17.974 × age. And for children aged 12–18 yrs was: FnNO = 579.222–30.332 × (male = 0, female = 1)—5.503 × age.

**Conclusions:**

Sex and age were two significant predictors of FnNO values for Chinese children(aged 12–18 yrs). Hopefully this study can provide some reference value for clinical diagnosis in children.

## Introduction

As a biomarker of airway inflammation, nitric oxide (NO) is a hot research topic in recent years. It is an important factor in inflammatory response, signal transduction, vasomotor and immune regulation (1). The upper airway is the main productive site of exhaled NO (2). Studies on healthy adults have shown that NO in the upper airway is mainly produced by epithelial cells in the nasal cavity, paranasal sinuses and nasopharynx. Concentration of NO in the paranasal sinuses is much higher than that in the nasal cavity and nasopharynx (3, 4). Fractional concentration of nasal NO (FnNO) test is to measure the NO produced by nasal cavity and sinuses, which has great value for the diagnosis of rhinitis and sinusitis and the assessment of the disease severity (5).

The normal values of FnNO (Nasal exhalation of nitric oxide) is within a normal range, which is different from FeNO (Fractional exhaled nitric oxide). Increased FnNO values due to inflammation suggest allergic rhinitis, and decreased FnNO values due to obstruction of sinus ostia suggest CRS (Chronic Rhinosinusitis) with nasal polyps (CRSwNP). For patients with extreme low FnNO values, the possibility of PCD (primary ciliary dyskinesia) and CF should be considered (6). At present, the consensus and guidelines of European and American recommend FnNO for the screening of PCD (7, 8), in which ciliary function disorder leads to obvious sinusitis. The Chinese guidelines for the diagnosis and treatment of allergic rhinitis also recommended FnNO as a part of the diagnosis of allergic rhinitis (9). Although there are many studies about normal values of FnNO, due to different test measures, the results are quite different.

The morbidity of rhinitis in China is extremely high, reaching 15.79% (10). And with the increasing attention of children with wet cough, diagnose of rhinitis and sinusitis is becoming urgent. At present, there is no consensus on the normal values of FnNO in children. The aim of this study is to obtain the normal values of FnNO in Chinese children aged 6–18 years, so as to provide reference for clinical diagnosis.

## Materials and methods

### Subjects

Twelve centers from 10 provinces of four regions (North China, East China, central China, and Northwest China) participated in the study. Each center recruited 400 healthy children, who were from local primary and secondary schools, and divided them into two age groups: group I (age: 6–11 years old, 100 males and 100 females respectively) and group II (age: 12–18 years old, 100 males and 100 females respectively). A questionnaire was designed to select qualified participants. The questionnaire included age, ethnicity, history of upper respiratory tract infection in the past 4 weeks, history of recurrent respiratory tract infection, history of allergic diseases such as asthma and rhinitis, and whether family members smoked in the home environment.The study was conducted with the informed consent of the children and their parents. The children were enrolled from February 2017 to June 2019.

The inclusion criteria were as follows: ① age: 6–18 years old; ② born in China with Chinese parents; ③ normal development of intelligence, can cooperate with the examination; The exclusion criteria were as follows: ① history of respiratory tract infection within 4 weeks; ② history of recurrent respiratory tract infection; ③ history of asthma, rhinitis and other allergic diseases; ④ family history of asthma or rhinitis; ⑤ smoking in the home; ⑥ deformities were found in the nose and face.The Ethical Committee of Shanghai Children's Medical Center approved the study protocol (SCMCIRB-K2017007), each sub-center follows the master research unit ethics. Clinical Trial:ChiCTR: 1800019029.

### Nasal No measurement

In this experiment, nitric oxide detection instrument is a chemiluminescence analyzer, which is also an internationally recognized laboratory technology for the determination of NO gold standard. FnNO was measured using the Nano Coulomb Breath Analyzer (Sunvou-CA2122, Wuxi, China) according to the American Thoracic Society/European Respiratory Society (ATS/ERS) recommendations (5, 11). Children were restrained from eating and drinking, strenuous exercise, and pulmonary function test within 1 h before the FnNO test.

Before the test, the operator explained and made sure that the children fully understood the procedures of the measurement. During the test, the children were asked to place a nasal olive firmly against one nostril and keep the other nostril open. After a deep inhalation, the children were asked to hold the breath for 10 s, while the air drawn through the olive at the flow rate of 10 ml/s into the analyzer.

To ensure the accuracy of the instrument, standard sample gas with NO concentration of 60 ppb and 250 ppb were used to calibrate the instrument before the start of the study in each center.

### Other measurements

The height and weight of all participants were measured. When measuring the height of children, the children are barefoot, and the height is accurate to 0.5 cm. When measuring the weight, children wear light clothes, and the weight is accurate to 0.1 kg.

### Statistical analysis

SPSS22.0 was used to analyze the results. Kolmogorov-Smirnov method was used to test the normality. As FnNO is normally distributed, the 95% percentile range (2.5%, 97.5%) were calculated as the normal range of FnNO values.

We performed multiple linear regression with age, sex, height, BMI, region as independent covariates to assess the significant predictors of FnNO. Weight was not included in the regression model because of multicollinearity with BMI. All *P* values were two-sided, and *P* < 0.05 was considered statistically significant.

## Results

### Study population

**Figure 1:**
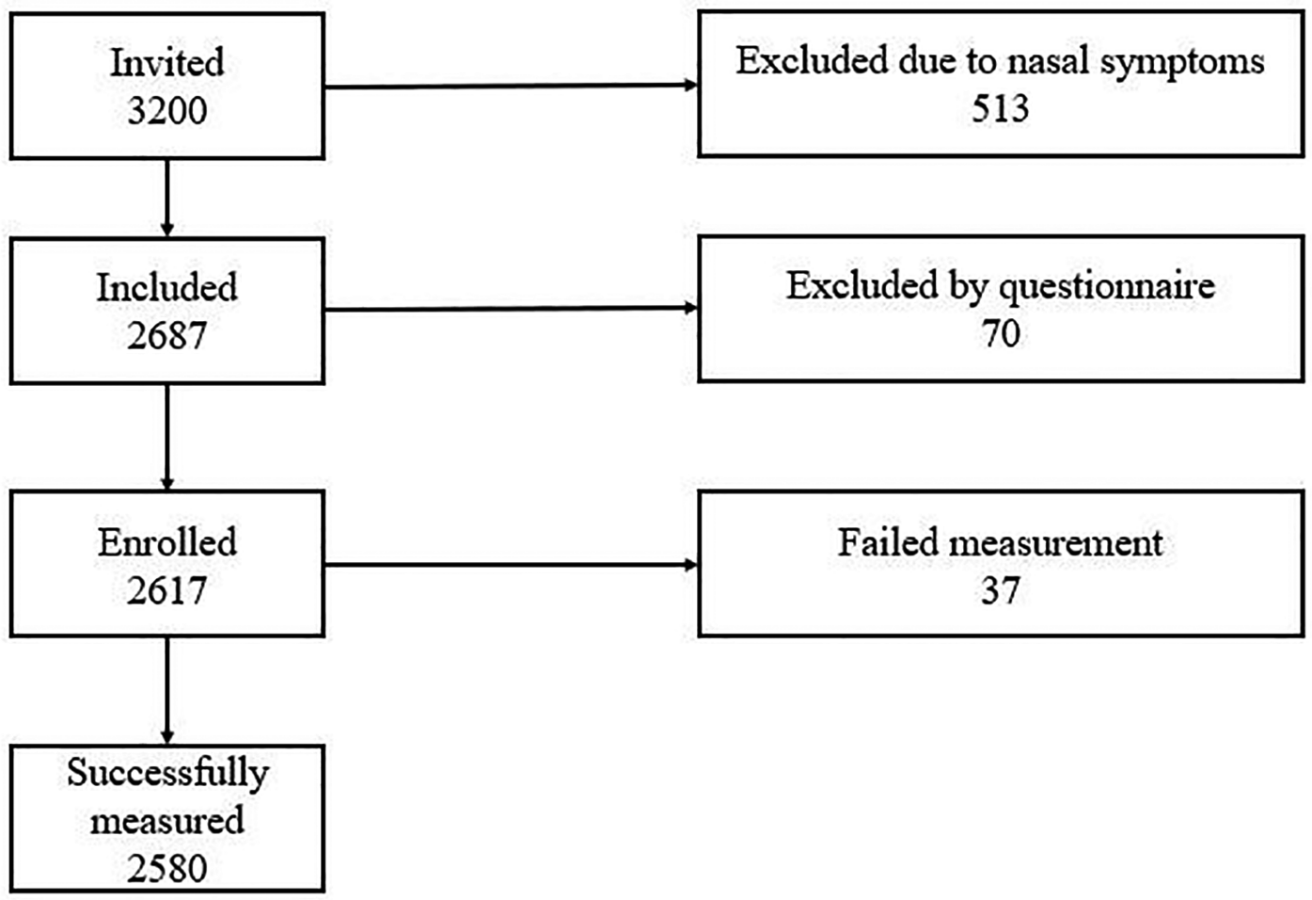
Flow chart of the study.

A total of 3,200 children were recruited into the study. Among them, 70 were beyond the required age range, 513 had upper respiratory symptoms, and 37 failed the measurement (Figure 1). Finally, 2,580 children (1,359 males and 1,221 females) were included in the analysis. There were 1,193 children (655 males and 538 females) aged 6–11 years and 1,387 children (704 males and 683 females) aged 12–18 years (Table 1).

**Table 1:** General characteristics of subjects.

	ALL	6–11 yrs	12–18 yrs
Number	2580	1193	1387
Male(%)	52.67%	54.90%	50.76%
Female(%)	47.33%	45.10%	59.84
Age (yrs)	11.9 ± 3.4	8.7 ± 1.7	14.6 ± 1.8
Height (cm)	152.0 ± 17.6	136.9 ± 11.9	165.0 ± 9.3
Weight (kg)	46.5 ± 17.1	33.6 ± 10.4	57.6 ± 13.4
BMI (kg/m^2^)	19.4 ± 4.0	17.5 ± 3.2	21.0 ± 3.9

### Predictors of fnNO

In children aged 6–18 years, FnNO is associated with age. In children aged 6–11 years, FnNO was positively associated with age, while in children aged 12–18 years, FnNO was negatively associated with age.. FnNO was not correlated with weight and BMI (*p* = 0.081, *p* = 0.100). Males had higher FnNO values than females (*p* = 0.017). There were differences in FnNO values among the four regions (*p* = 0.000). On multivariate analysis, only female sex, height and age were predictors of FnNO (*r* = −14.795, *p* = 0.039; *r* = 1.344, *p* = 0.001; *r* = −5.056, *p* = 0.016).

**Figure 2:**
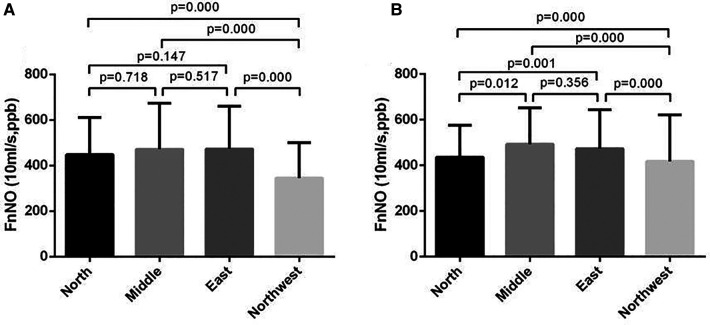
Fnno values of children from four regions of China. (**A**): FnNO values of children aged 6–11 yrs; (**B**): FnNO values of children aged 12–18 yrs.

**Figure 3:**
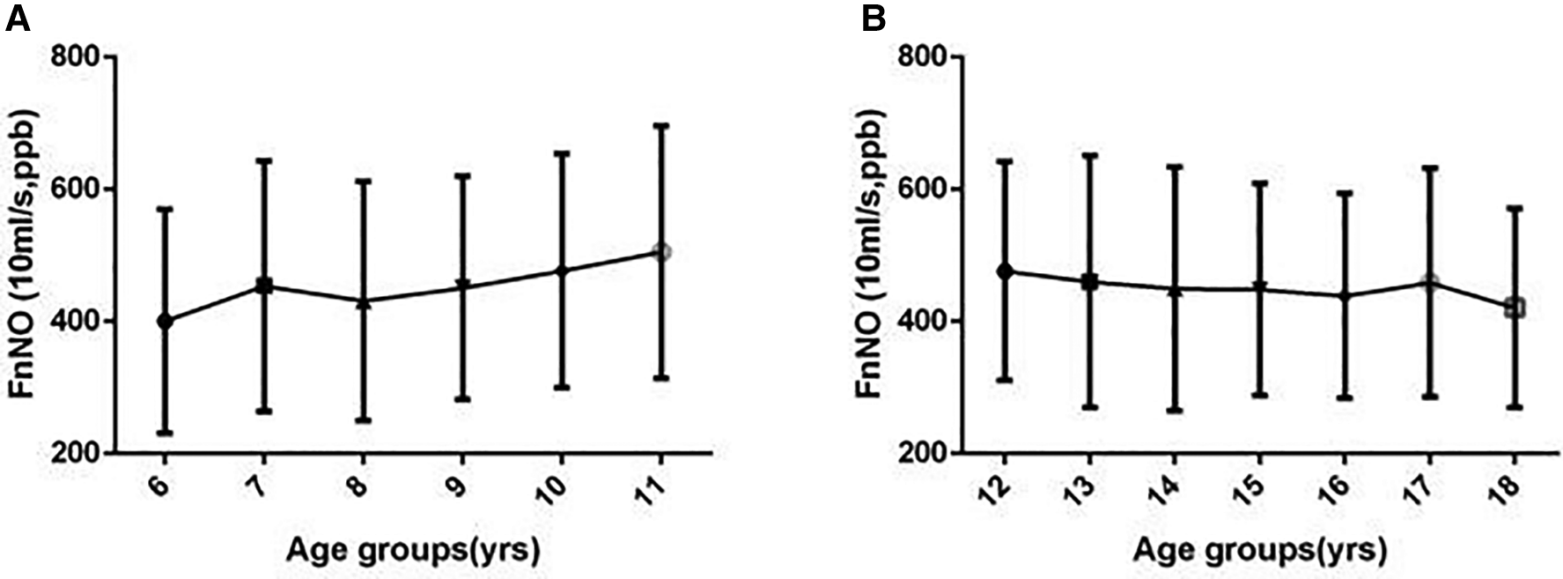
Fnno values in children by age. (**A**) FnNO values increased with age in children aged 6–11 yrs; (**B**) FnNO values decreased with age in children aged 12–18 yrs.


For children aged 6–11 yrs, FnNO was positively correlated with age (*r* = 13.936, *p* = 0.013). FnNO was not correlated with height, weight and BMI (*p* = 0.438, *p* = 0.693, *p* = 0.943). There was no significant difference between male and female children (*p* = 0.154). There were differences in FnNO values among the four regions (*p* = 0.000, Figure 2A). On multivariate analysis, age was the only predictor of FnNO (*r* = 17.974, *p* = 0.000, Figure 3A). The prediction equation of FnNO values for children aged 6–11 yrs was:

FnNO=298.881+17.974×Age

For children aged 12–18 yrs, FnNO was negatively correlated with age (*r* = −8.750, *p* = 0.001), and was not correlated with height, weight or BMI (*p* = 0.098, *p* = 0.234, *p* = 0.186). Males had higher FnNO values than females (*p* = 0.001). There were differences in FnNO values among the four regions (*p* = 0.000, Figure 2B). On multivariate analysis, female sex and age were predictor of FnNO (*r* = −30.332, *p* = 0.001; *r* = −5.503, *p* = 0.027; Figure 3B). The prediction equation of FnNO values for children aged 12–18 yrs was:

FnNO=513.186−30.332×(male=0,female=1)−5.503×(age−12)

### Normal values of fnNO

**Figure 4:**
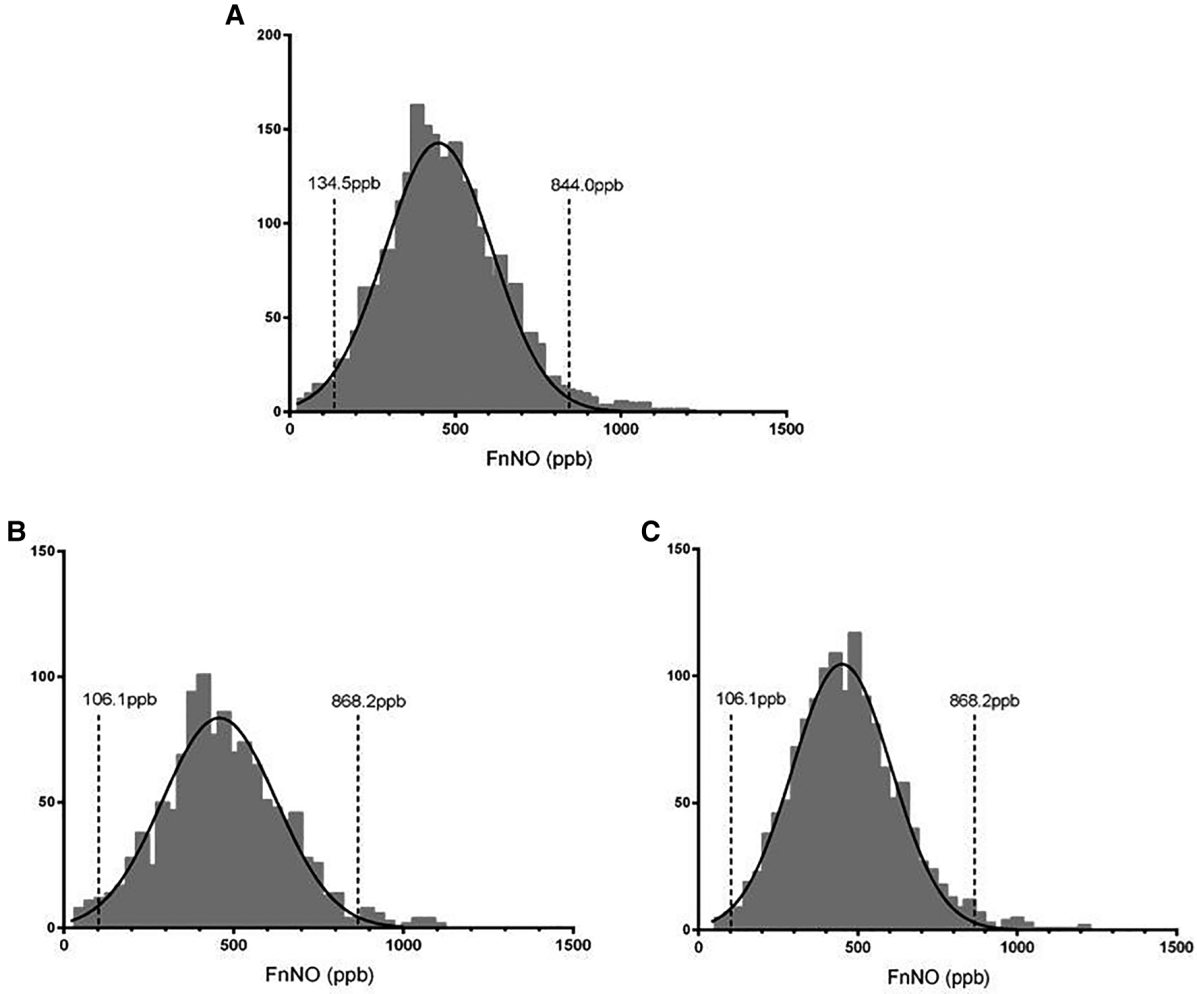
Distribution of fnNO values in children. (**A**) FnNO values of children aged 6–18 yrs; (**B**) FnNO values of children aged 6–11 yrs; (**C**) FnNO values of children aged 12–18 yrs.

In this study, FnNO ranged from 26 to 1191 ppb for children aged from 6 to 18 yrs. FnNO values were normally distributed (mean: 454.5 ppb, SD: 176.2 ppb), and the 2.5th to 97.5th percentile values were 134.5–844.0 ppb (Figure 4A). Among them, the average FnNO value for children aged 6–11 yrs was 455.5 ppb (SD: 181.7 ppb), and the 2.5th to 97.5th percentile values were 106.1–868.2 ppb (Figure 4B). The average FnNO value for children aged 12–18 yrs was 453.7 ppb (SD: 171.4 ppb), and the 2.5th to 97.5th percentile values were 149.0–842.6 ppb (Figure 4C).

## Discussion

In the respiratory system, NO is mainly involved in immune defense, airway tension, mucosal blood flow, ciliary movement and regulation of glandular secretion (5). NO in the nasal cavity can be antiviral, inhibit the proliferation of sinus bacteria, regulate inflammation, increase the swing of cilia, promote the discharge of microorganisms, together constitute the nasal defense barrier, can maintain the relatively sterile sinus. Clinically, elevated nNO levels may indicate acidophilic granules in the nasal cavity.

Cellular inflammation, or no significant obstruction of the sinuses. For example, after standardized treatment with nasal glucocorticoids, nNO concentration decreased even to a normal level, which can also indicate that nasal inflammation is better than before.

The FnNO values of 2,580 children aged 6–18 yrs were measured in this study. The results showed that the FnNO values were normally distributed, and the FnNO values of 95% of children ranged from 134.5 ppb to 884.0 ppb. For children under 12 years old, FnNO value was positively correlated with age. For children over 12 years old, FnNO values were negatively correlated with age, and males had higher FnNO values than females.

Normal value of FnNO in children have been studied in some previous research (12–20). The sample size ranged from 19 to 436 subjects. Different studies have different conclusions on the range of normal value and related influencing factors (see Table 2). Among them, three studies have studied the FnNO values of Chinese population, and the results are inconsistent (12–14). This study excluded subjects with the history of upper respiratory tract infection, recurrent respiratory tract infection, asthma, rhinitis, and other allergic diseases. Sex, age, height, weight, BMI, region were all analyzed for predicting factors.

**Table 2:** Reported fnNO values and predictors.

Year	First author	Healthy people	Age (yrs)	Flow rate (ml/s)	FnNO levels in ppb	FnNO associated
2005	Edwards EA (17)	*N* = 139	5–17	8.3	Median(range): 403 (34–1120)	
2005	Struben VM (15)	*N* = 340	6–17	11.7	Mean ± SD: 449 ± 115	With age (<12 yrs), but not with gender, height, weight, or BMI
2009	Zhang L (12)	*N* = 80	18–44	5.3–5.4	Mean ± SD: 819 ± 211	With age and gender, but not with height or BMI
2010	Piacentini GL (16)	*N* = 43	3–7	5	Mean ± SD: 396 ± 25	With age and weight, but not with gender, height, BMI
2011	Marthin JK (18)	*N* = 30	3.1–63.6	5	Mean ± SD: 788 ± 41	
2011	Mateos-Corral (19)	*N* = 19	5–18	5.5	Mean ± SD: 1193.2 ± 374.3	
2012	Leng G (13)	*N* = 182	18–76	5	Mean ± SD:79 ± 35	Not with age, gender, height, BMI
2016	You S (14)	*N* = 436	9–22	5	Mean ± SD: 273.5 ± 112.3	Not with age, gender, height, weight, BMI and BSA
2017	Menou A (20)	*N* = 31	6–16	5	Mean ± SD:659.7 ± 231.5@@ Range: 307–1330	Not with age, gender, height, weight, BMI and BSA

The results showed that age had a correlation with FnNO values of children under 12 years old and over 12 years old. For children under 12 years old, there was a positive correlation between age and FnNO. Multiple regression analysis showed the prediction rules was: FnNO = 298.881 + 17.974 × age. Which means for children less than 12 years old, FnNO increased by 18 ppb with age increasing by 1 year. This was similar to the results of Struben et al. In their study, it was found that the FnNO values of children under 12 yrs old increased by 11.5 ppb with each increase of one year (15). The positive correlation between FnNO and age may be related to the development of paranasal sinuses. The concentration of NO in paranasal sinuses is significantly higher than that in nasal cavity. Therefore, with the increase of age, the development of paranasal sinuses is gradually complete, and FnNO increases (21, 22). Another reason may be adenoid atrophy. Adenoids develop in infancy and reach the maximum size at 2–14 years old. With the development of immune system, adenoids started to degenerate. Adenoid atrophy leads to the increase of airway area in nasopharynx, which increases the FnNO values (23). For children over 12 years old, there was a negative correlation between age and FnNO values (*r* = −5.503, *P* = 0.027), which was also close to the results of Struben's study，but the specific reasons for the negative correlation with age need to be further studied. It is possible that in children over 12 years old, the sinuses develop and mature, and as they grow older, they become more and more close to the adult normal..However,Struben's study (15) and our study all showed that the affection caused by various factors was limited, which could be ignored comparing to the wider range of FnNO values.

For children over 12 years old, males had higher FnNO values than females, and there was a negative correlation between female gender and FnNO values (*r* = −30.332, *P* = 0.001). Many studies have found that males had higher FeNO values than females (both adults and children). This is mainly due to the larger airway surface area and diameter in men than in women, resulting in different diffusion of NO (24). However, there are fewer studies on FnNO values. Zhang l et al. (12) found that male adults had higher FnNO values than female adults. One of the reasons is that the height of men is generally higher than that of women, so the airway cavity of women is smaller than that of men (25). Another reason may be the respiratory rate and basal metabolic rate of male and female are different.

When children aged 6–11 yrs and 12–18 yrs were analyzed, it was found that regional differences also affect FnNO values. The FnNO values in the North of China, especially in the Northwest, were lower than that in the East and Central China. Previous study had shown that there were differences in FeNO values in different regions of China, but no studies had reported the impact of regional differences on FnNO values. The impact of regional1 differences may be due to the differences of environment and diet in different regions. In addition, the different seasons of study time in different regions may also be the reason. It was found that FnNO values were more affected than FeNO values for different factors such as the season (26) and time of measurement (27). For children with allergic rhinitis, the level of FnNO increased during pollen season (28). In addition, the air quality and environmental pollution in different areas were different, and the FnNO values may also be different. Studies had found that the NO metabolites in nasal lavage fluid of urban children was higher than that of rural children, which may be related to environmental pollution (29).

There were many studies on the normal values of FnNO in normal adults and children, but the ranges obtained from different studies were quite different (12–20) (Table 2). This was mainly due to the different flow rates of analyzers used in these studies, ranging from 5 ml/s to 11.7 ml/s. FnNO is flow rate dependent, the greater the flow rate, the smaller the FnNO value will be (5). In this study, a flow rate of 10.0 ml/s was used to aspirate gas into analyzer. The results showed that the FnNO values of children aged 6–18 was 454.5 ± 176.2 ppb, which was close to the results of Struben et al. (15). In their study, 340 children were studied, using the nasal aspiration flow rate of 700 ml/min (11.7 ml/s). The results showed that the mean FnNO values of 6–17 years old children was 449 ± 115 ppb. The sampling rate in this study was close to that in Struben's study, so the mean FnNO values were also remarkably close, which had a certain clinical reference value.

According to the European position paper on diagnostic tools in rhinology published in 2019, the measurement at the flow rate of 700 ml/min (approximately 10 ml/s) was more reproducible. The range of 300–500 ppb was recommended as the normal FnNO values (6). In our study, the FnNO values of children aged 6–18 yrs was 454.5 ± 176.2 ppb, which was higher than the recommended normal range. This may be because the normal children in this study only passed the questionnaire survey and did not carry out blood IgE screening, which could not completely exclude the children with allergic diseases.

In conclusion, sex and age (only for children aged 12–18 yrs) were two predictors of FnNO values for children. The mean FnNO values of Chinese aged 6–18 yrs was 454.5 ± 176.2 ppb, and 95% of them were in the range of 134.5–844.0 ppb. The prediction rule of FnNO values for Chinese children aged 6–11 yrs was: FnNO = 298.881 + 17.974 × age. And for children aged 12–18 yrs was: FnNO = 579.222–30.332 × (male = 0, female = 1)—5.503 × age. Hopefully this study can provide some reference value for clinical diagnosis in children.

However, current detection methods are limited to the direct detection of NO concentration in the anterior nostril, which is insufficient to truly reflect the distribution and diffusion of NO in the nasal cavity and sinuses. Therefore, nNO as a marker of nasal inflammation still needs to be further studied.

The authors declare that the research was conducted in the absence of any commercial or financial relationships that could be construed as a potential conflict of interest.

## Data Availability

The original contributions presented in the study are included in the article/Supplementary Material, further inquiries can be directed to the corresponding author.
